# High diversity of protistan plankton communities in remote high mountain lakes in the European Alps and the Himalayan mountains

**DOI:** 10.1093/femsec/fiv010

**Published:** 2015-01-28

**Authors:** Barbara Kammerlander, Hans-Werner Breiner, Sabine Filker, Ruben Sommaruga, Bettina Sonntag, Thorsten Stoeck

**Affiliations:** 1 University of Innsbruck, Institute of Ecology, Lake and Glacier Research Group, Technikerstrasse 25, 6020 Innsbruck, Austria; 2 University of Innsbruck, Research Institute for Limnology, Mondsee, Ciliate Ecology and Taxonomy Group, Mondseestrasse 9, 5310 Mondsee, Austria; 3 University of Kaiserslautern, Department of Ecology, Gottlieb-Daimler-Strasse Building 14, 67663 Kaiserslautern, Germany

**Keywords:** diversity, alpine lakes, next-generation sequencing

## Abstract

We analyzed the genetic diversity (V4 region of the 18S rRNA) of planktonic microbial eukaryotes in four high mountain lakes including two remote biogeographic regions (the Himalayan mountains and the European Alps) and distinct habitat types (clear and glacier-fed turbid lakes). The recorded high genetic diversity in these lakes was far beyond of what is described from high mountain lake plankton. In total, we detected representatives from 66 families with the main taxon groups being Alveolata (55.0% OTUs_97%_, operational taxonomic units), Stramenopiles (34.0% OTUs_97%_), Cryptophyta (4.0% OTUs_97%_), Chloroplastida (3.6% OTUs_97%_) and Fungi (1.7% OTUs_97%_). Centrohelida, Choanomonada, Rhizaria, Katablepharidae and Telonema were represented by <1% OTUs_97%_. Himalayan lakes harbored a higher plankton diversity compared to the Alpine lakes (Shannon index). Community structures were significantly different between lake types and biogeographic regions (Fisher exact test, *P* < 0.01). Network analysis revealed that more families of the Chloroplastida (10 vs 5) and the Stramenopiles (14 vs 8) were found in the Himalayan lakes than in the Alpine lakes and none of the fungal families was shared between them. Biogeographic aspects as well as ecological factors such as water turbidity may structure the microbial eukaryote plankton communities in such remote lakes.

## Abstract (precis)

Diversity of protistan communities in remote high mountain lakes in the European Alps and the Himalayan mountains suggests impact of glacier retreat on community structure and function.

## Graphical abstract

**Graphical Abstract Figure.**
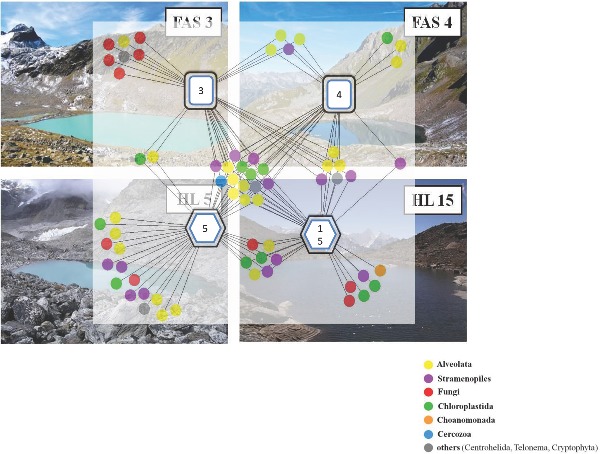
Diversity of protistan communities in remote high mountain lakes in the European Alps and the Himalayan mountains suggests impact of glacier retreat on community structure and function.

## INTRODUCTION

In the last years, molecular tools have seized biodiversity studies, and next-generation sequencing (NGS) approaches have become popular instruments to estimate protist diversity (Margulies *et al.*, 2005). To date, most studies on microbial diversity including protists and fungi have focused on marine ecosystems (e.g. Alexander *et al.*, 2009; Stoeck *et al.*, 2010; Edgcomb *et al.*, 2011; Bik *et al.*, 2012; Stock *et al.*, 2012). Even though the diversity of freshwater microbial plankton is presumably much higher than in the marine environment (Logares *et al.*, 2009; Auguet, Barberán and Casamayor 2010; Barberán and Casamayor 2010; Barberán *et al.*, 2011; Triadó-Margarit and Casamayor 2012), only few freshwater lakes were examined so far by using such molecular tools (e.g. Chen *et al.*, 2008; Lefèvre *et al.*, 2008; Steele *et al.*, 2011; Charvet *et al.*, 2012a; Charvet, Vincent and Lovejoy 2012b; Charvet, Vincent and Lovejoy 2014; Stoeck *et al.*, 2014). Hitherto only very few sequence data are available from protistan plankton in high mountain lakes (e.g. Triadó-Margarit and Casamayor 2012). Yet, these data emphasized high mountain lakes as diversity hotspots for (hitherto unknown) eukaryotic microbial plankton. Such a scarce knowledge on microbial eukaryotic plankton in high mountain lakes is unsatisfying considering the ecological importance of these organisms in the energy and carbon transfer within aquatic food webs (e.g. Azam *et al.*, 1983; Sherr and Sherr 1988; Weisse and Müller 1998; Sonntag *et al.*, 2006; Zingel *et al.*, 2007).

Likewise, microscopy studies on protist diversity in high mountain lakes are scarce (Félip *et al.*, 1999; Straškrabová *et al.*, 1999; Wille *et al.*, 1999; Sonntag, Summerer and Sommaruga 2011). The difficulties that occur in protist investigation by microscopy are their small sizes, few morphological characters available to identify especially flagellated species and low abundance particularly in high mountain lakes. Though, as protists include manifold groups that have specific demands on their environment concerning their nutrition or temperature, there is a need to identify them as exact as possible to obtain an overall picture of the food web interactions. To overcome such inconveniences, NGS approaches such as pyrosequencing (Margulies *et al.*, 2005) are promising techniques to unravel the hidden diversity of microbes in environmental samples (Sogin *et al.*, 2006; Caron *et al.*, 2012). One major strength of NGS is the depth of sequencing which allows elucidating the local ‘rare biosphere’ (Sogin *et al.*, 2006), a seed bank of low abundant taxa that may play a pivotal role in ecosystem response to environmental changes as well as in ecosystem stability and function(ing) (Pedrós-Alió 2007; Dawson and Hagen 2009; Stoeck and Epstein 2009).

In high mountain lakes organisms are confronted with short growing seasons, low food availability or high incident solar radiation (Sommaruga 2001; Rose *et al.*, 2009). Characteristically, high mountain lakes can be very transparent or turbid dependent on the connection to a glacier. The lower the particle load and the concentration of chromophoric dissolved organic matter, the more transparent are such lakes. Lake transparency is crucial for the biota because it determines the penetration of photosynthetically active radiation and of ultraviolet radiation (UVR). For example, in clear high mountain lakes the potentially harmful wavelengths of UVR can reach down to the lake bottom (Morris *et al.*, 1995; Laurion *et al.*, 2000; Sommaruga and Augustin 2006). As for clear high mountain lakes, several surveys have concentrated on the effects of UVR onto the planktonic community and their key players, but it is largely unknown how sensitive species from turbid alpine lakes are to UVR. However, a recent comparative study with one copepod species living in clear and turbid alpine lakes suggests that photoprotection and DNA repair mechanisms are important in adapting to the shift in water transparency (Tartarotti et al., 2014).

In our study, we sampled four different lakes, including two biogeographic regions (Austrian Central Alps and the Himalayan mountains, Nepal) and two lake types (clear vs glacier-fed turbid lakes) using massively parallel tag sequencing (pyrosequencing) of the hypervariable V4 region of the small subunit ribosomal DNA to determine the plankton diversity. The reasoning for these habitat choices is among others to maximize the extent of diversity that can be identified from different mountain ranges and different lake types. Our data shows a much higher diversity of fungal and protistan plankton as known from previous diversity studies in high mountain lakes, with ca. 60% of our detected sequences showing a high genetic divergence to deposited sequence data.

## MATERIALS AND METHODS

### Study sites, sampling and analyses

#### Faselfad lakes

**Figure 1.**
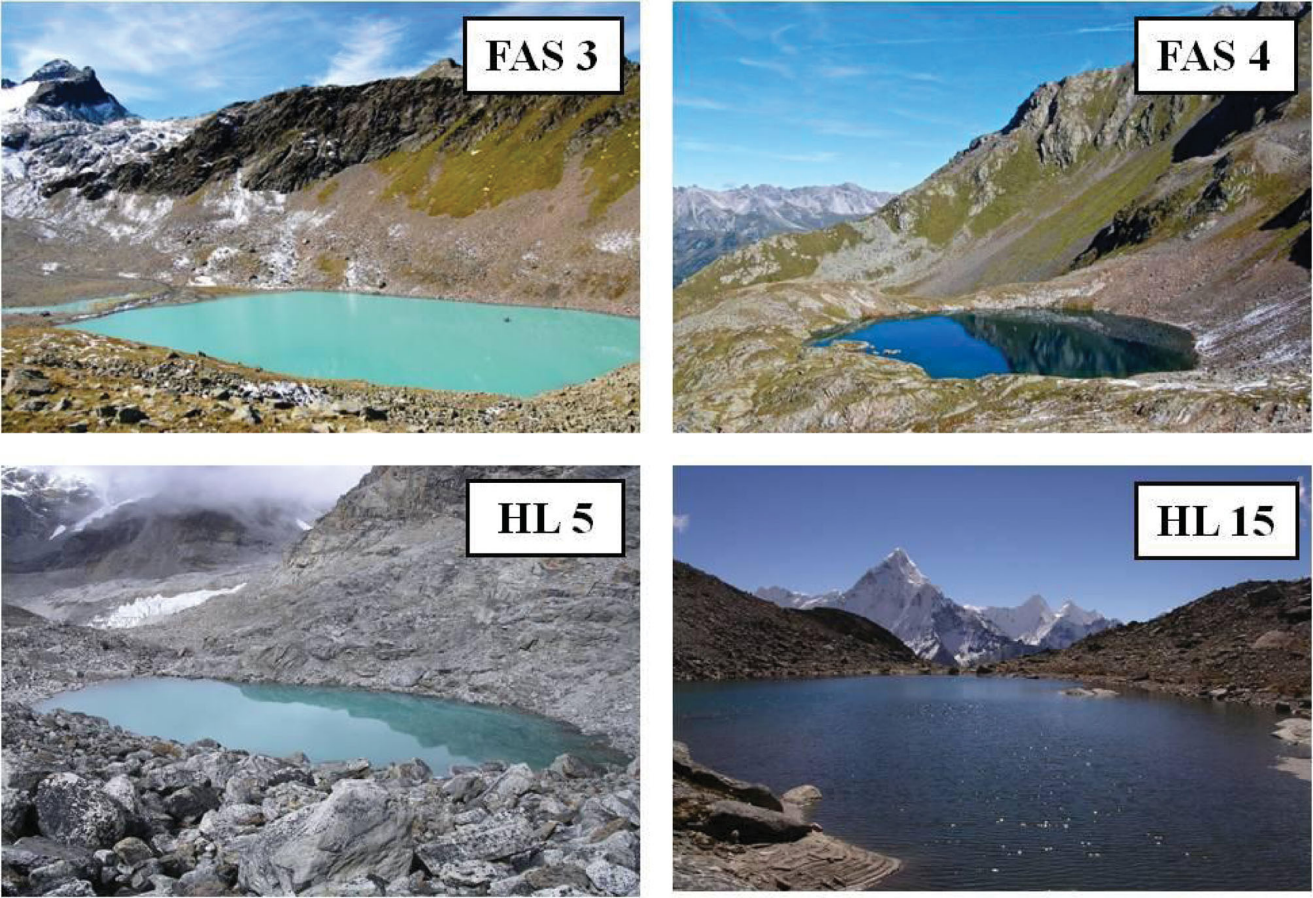
The studying sites FAS lakes and HL lakes: FAS 3 is located about 200 m below the glacier at 2414 m a.s.l. and the clear lake FAS 4 at 2416 m a.s.l. HL 5 (5400 m a.s.l.) and HL 15 (5160 m a.s.l.) are located in the Khumbu Valley. Photos from T. Stoeck (FAS) and R. Sommaruga (HL).

The study site Faselfad (FAS) is located in the western Austrian Central Alps and comprises a group of six adjacent lakes situated between 2263 and 2620 m above sea level (a.s.l.). All six lakes originate from one glacier, the ‘Faselfadferner’, and mainly differ in altitude and water transparency. Out of these, we selected one clear (FAS 4) and one glacier-fed turbid lake (FAS 3) (Fig. 1, Table 1). FAS 3 is located approximately 200 m below the glacier and fed by glacial melt water enriched with high particle loads, so-called ‘glacial flour’ and partially by water from the catchment. The clear lake FAS 4 has lost its connectivity to the glacier and is fed by seepage from its catchment (Sommaruga and Kandolf 2014).

**Table 1.** Geographic (latitude, longitude and altitude) and lake characteristics of the FAS and the HL lakes: maximum depth (Z_max_), area, abiotic parameters (min-max; mean) such as turbidity, conductivity, pH, TP, DOC, nitrate (NO_3_-N) and ammonium (NH_4_-N). *values only available from surface water; n.d. not determined. (Himalaya data from Sommaruga and Casamayor, 2009; FAS data: for additional measured parameters, see Tartarotti *et al.*, 2014).

Region	Lake	Latitude	Longitude	Altitude	Z_max_	Area	Turbidity & visual/optical appearance	Conductivity	pH	TP	DOC	NO_3_-N	NH_4_-N
				(m a.s.l.)	(m)	(km^2^)	(NTU)	(μS cm^−1^)		(μg L^−1^)	(μg L^−1^)	(μg L^−1^)	(μg L^−1^)
European Alps	FAS 3	47°N 04^′^ 15^′^	10°E 13^′^ 15^′^^′^	2414	17.0	0.02	5.71–11.80; 8.57 turbid	42.0–45.8; 43.2	7.2–8.8; 8.0	7.3–10.6; 8.8	220.0–318.0; 266.9	89.0–201.0; 127.9	1.0–4.0; 1.7
	FAS 4	47°N 04^′^ 27^′^^′^	10°E 13^′^ 34^′^^′^	2416	15.0	0.02	0.02–0.26; 0.17 transparent	48.8–51.5; 49.8	7.2–7.4; 7.3	1.7–5.7; 2.5	268.0–374.0; 305.1	158.0–165.0; 162.3	1.0–2.0; 1.1
Himalaya	HL 5	27°N 59^′^ 45^′^^′^	86°E 49^′^ 24^′^^′^	5400	4.0	0.01	n.d. turbid	32.1–32.4; 32.3*	7.2–7.2;7.2*	3.0–44.0; 3.7*	270.0–830.0; 550.0*	20.0–22.0; 21.3*	5.0–7.0; 5.7*
	HL 15	27°N 56^′^ 34^′^^′^	86°E 47^′^ 40^′^^′^	5160	4.8	0.01	n.d. transparent	57.3–57.5; 57.4	6.7–6.7; 6.7	0.0–1.0; 0.7	310.0–450.0; 363.3	40.0–42.0; 40.7	4.0–6.0; 6.7

On 29 August 2011, water samples were collected with a 5-L Schindler–Patalas sampler at the deepest point of each lake from an inflatable boat. Mixed water samples (10 L) in a ratio of 1:1:1 were taken from the uppermost meters (0, 1 and 2 m) and 1 m above and below the chlorophyll *a* (chl *a*) maximum. This sampling strategy was based on previous samplings along vertical depth gradients indicating that most of the taxa were found around the chl *a* maximum (Kammerlander *et al.*, unpublished). Prior to sampling, the chl *a* maximum in the water column was detected with a Backscat I-Fluorometer (Haardt, model 1101.1, excitation 380–540 nm, emission 685 nm). Further, subsamples were taken for the analysis of abiotic parameters, i.e. turbidity, conductivity, pH, total phosphorus (TP), dissolved organic carbon (DOC), nitrate (NO_3_-N) and ammonium (NH_4_-N). The chemical parameters were measured in the laboratory of the Institute of Ecology at the University of Innsbruck as described in Sommaruga-Wögrath *et al.* (1997). For measuring the turbidity a handheld turbidimeter (WTW Turb 430 T) was used. For details, see Sommaruga and Kandolf (2014). The DOC analyses were measured with a high temperature catalytic oxidation method (Shimadzu TOC-V_CPH_-total organic carbon analyzer). For details, see Laurion *et al.* (2000) and Sommaruga and Augustin (2006). For nucleic acid extractions, triplicate water samples were collected in clean plastic carboys and 2–3 L each was drawn onto Durapore membranes (0.65 μm, 47 mm, Millipore) using a peristaltic pump. Filters were frozen immediately in liquid nitrogen. Samples were stored at −20°C until DNA extraction.

#### Himalaya lakes

One glacier-fed turbid (HL 5) and one clear (HL 15) lake were sampled in the Khumbu Valley region (Nepal) close to Mount Everest on 8 and 9 October 2004 (Fig. 1, Table 1). For more details on this study site, see Tartari *et al.* (1998) and Sommaruga and Casamayor (2009).

Samples were collected from a boat and integrated over the water column. Filters for nucleic acid extraction were prepared as described above and transported to Innsbruck in liquid nitrogen.

### Pyrosequencing

#### DNA isolation and construction of pyro-amplicon libraries

DNA was isolated directly from the Durapore membranes using Qiagen's AllPrep kit according to the manufacturer's instructions. The samples (filters) were extracted and pooled. From these extracts, the hypervariable V4 region of the 18S rRNA gene was amplified using the eukaryote specific primer pair TAReukV4F and TAReukREV (5^′^-ACTTTCGTTCTTGATYRA-3^′^, Stoeck *et al.*, 2010) yielding ca. 500 basepair (bp) fragments. To distinguish the different samples in downstream processes, the V4 forward primer was tagged with specific 10-bp identifiers (MIDs) at the 5^′^-end. The PCR protocol followed the description of Stoeck *et al.* (2010). To minimize PCR-bias, we ran three individual reactions per sample. The resulting PCR products were purified (MinElute PCR purification kit, Qiagen, Germany) and pooled prior to sequencing. The V4-DNA amplicon libraries were sequenced on one-half of a PicoTiter Plate with a Roche FLX GS20 sequencer and the Titanium chemistry (FAS lakes: EnGenCore, SC, USA; HL lakes: LGC Genomics, Berlin, Germany).

#### V4-amplicon data processing

Amplicons were denoised with the software Acacia (Bragg *et al.*, 2012; http://sourceforge.net/projects/acaciaerrorcorr/). For further data cleaning, including chimera checking and data analyses, we used the software package QIIME (Caporaso *et al.*, 2010). After quality filtering, only reads with exact barcodes and primers, a quality score >25, unambiguous nucleotides and a minimum length of 300 bp were kept. The remaining sequences were then checked for chimeras and clustered at different threshold levels (90, 95, 96, 97, 98, 99 and 100%) using the OTUpipe script (Edgar *et al.*, 2011) implemented in QIIME. The OTUpipe script was done with the following adjustments: -m usearch –l –reference_chimera_detection –j 1 –s 0.xx –word_length yy. The word length value was calculated according to an equation given in Edgar *et al.* (2011). For our pyro-amplicons, a value of 64 was used. For taxonomic assignments, one representative sequence (longest) from each OTU was extracted and analyzed with the software package JAguc (Nebel *et al.*, 2011a) and GenBank´s nr nucleotide database release 187 as reference database. JAguc employs BLASTn searches, with algorithm parameters adjusted for short reads (-m 7 -r 5 -q -4 -G 8 -E 6 -b 50). Using a custom Java-based script, the output files from QIIME´s OTUpipe and JAguc were merged. Non-target OTUs (metazoans and embryophytes) were excluded and the resulting file converted into a biom file, which was then used as a basis for statistical and network analyses.

#### Statistical and network analyses

Rarefaction profiles and Shannon index (alpha-diversity), as well as Chao–Jaccard beta-diversity, were calculated in QIIME. For this purpose, data were normalized and resampled 1000 times to account for uneven sample sizes (Logares *et al.*, 2012). UPGMA (Unweighted Pair Group Method with Arithmetic mean)-clustering was used to construct Chao–Jaccard distance dendrograms.

A table including the number of observed OTUs (only amplicons were considered that were at least 95% similar to database entries) across each sample and their taxonomic assignment (rank: family) was generated and subjected to QIIME for calculation of a network data file. Cytoscape (Cline *et al.*, 2007) was used to visualize and analyze shared and exclusive families in samples. Nomenclature follows Adl *et al.* (2012). We created a network graph with an ‘edge-weighted spring embedded layout’ where every dot represented a taxonomic family, which was colored according to its phylogenetic affiliation. For more details, see http://qiime.org/tutorials/making_cytoscape_networks.html.

Additionally, Fishers exact tests (Fisher 1922) were run to test the non-random independency of the datasets among the lakes, i.e. the null hypothesis was that the taxon distribution was equal in all lakes. For this statistical analysis, we used the vegan package of R.

## RESULTS

### Lake characteristics

The turbidity in FAS 3 was 16-fold higher than in FAS 4 (Table 1). Though turbidity was not directly measured in HL 5 and HL 15, the optical appearance of both lakes was similar to the according turbid and clear FAS lakes (Sommaruga pers. obs., Fig. 1). The nitrate (NO_3_-N) values were generally higher in the Alpine lakes than in the Himalayan ones (mean NO_3_-N of Alpine lakes vs HL lakes: ∼145 vs 31 μg L^−1^).

In the clear lakes, conductivity and DOC concentrations were higher than in the turbid lakes and the concentrations of TP and the pH were lower in the clear lakes, with the highest TP concentration (mean TP: 8.8 μg L^−1^) observed in FAS 3 (Table 1).

### Overview of V4 amplicon data

After quality check, 226 267 sequences in total with >300 bp length (mainly between 350 and 450 bp) were used for the taxonomic assignments (Table 2; Fig. S1, Supporting Information). Our target organisms were eukaryotic unicellular organisms and fungi checked at least at the family level. Non-target sequences (1.4%), unassigned sequences (0.9%) and singletons/ doubletons were removed finally resulting in 219 155 sequences, and grouping into 1804 operational taxonomic units (OTUs) called at 97% sequence similarity. Different cluster thresholds (90–100%) were applied (Fig. S2, Supporting Information) and the rarefaction curves (Fig. S3, Supporting Information) showed that we obtained saturated sampling profiles for OTUs called at 97% sequence similarity. Approximately 60% of the target sequences had a best BLAST hit with >95% sequence similarity to a deposited sequence of a described taxon (Fig. S4, Supporting Information), indicating that a relatively large proportion of the data points to an as yet unsequenced novel diversity in high mountain lakes.

**Table 2.** Number of sequences and OTUs generated after 454 data processing with 97% cluster threshold: target sequences = eukaryotic unicellular organisms and fungi, checked at least at the family level. Non-targets = multicellular organisms such as higher plants (Embryophyta) and Metazoa, prokaryotes and unassigned sequences.

	Total reads	After QIIME quality check	Target sequences	Target sequences without singletons/doubletons	Non-target sequences	Unassigned sequences
Sequences	350 803	226 267	221 011	219 155	3241	2015
OTUs		3252	3073	1804	118	61

### Plankton diversity and partitioning of diversity

**Figure 2.**
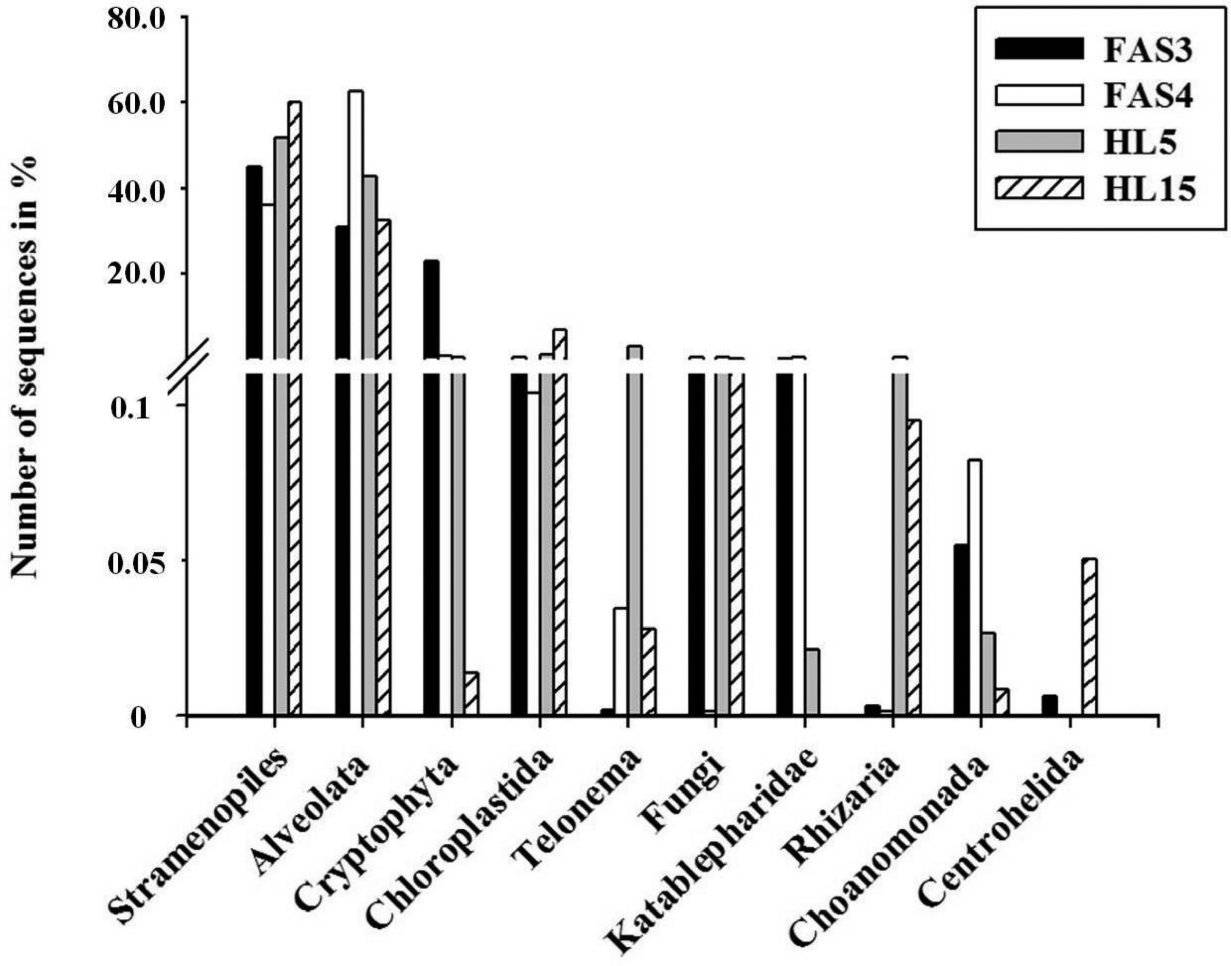
Distribution of the protistan and fungal communities among the lakes (expressed in number of sequences in% per lake; Faselfad lakes, FAS; Himalaya lakes, HL). Note that most of the sequences were assigned to the Alveolata (45.4% of total sequences), Stramenopiles (45.2%), Cryptophyta (6.8%) and Chloroplastida (1.5%). All other groups were <1% of the total sequences.

The main groups detected in all lakes belonged to the alveolates (55.0% of total OTUs_97_%), the stramenopiles (34.0% of total OTUs_97%_), the cryptophytes (4.0% of total OTUs_97%_), the chloroplastids (3.6% of total OTUs_97%_) and the fungi (1.7% of total OTUs_97%_). The contribution of the phyla Centrohelida, Choanomonada, Rhizaria, Katablepharidae and Telonema was <1% of the total OTUs_97%_. The number of total sequences followed the same ranking except for the fungi, which represented <1% of the total sequences (Fig. 2).

**Figure 3.**
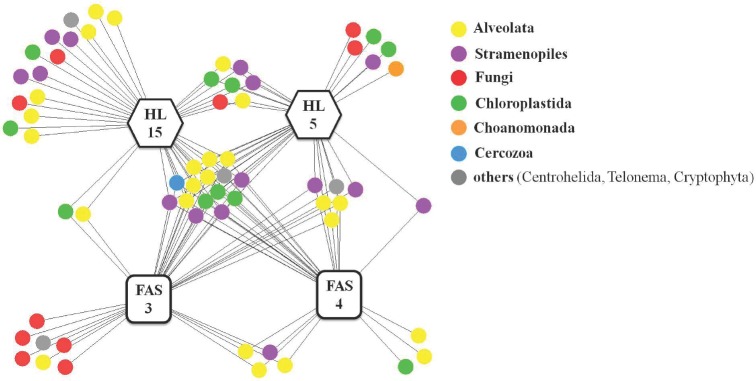
Network of the protistan and fungal communities in the glacier-fed turbid FAS 3 and the clear FAS 4 lake in the Austrian Alps and the glacier-fed turbid HL 5 and clear HL 15 lake in the Himalayan mountains using Cytoscape (version 2.8.3). Each dot represents a taxonomic family, which was colored according to its phylogenetic affiliation; Faselfad lakes, FAS; Himalaya lakes, HL.

**Figure 4.**
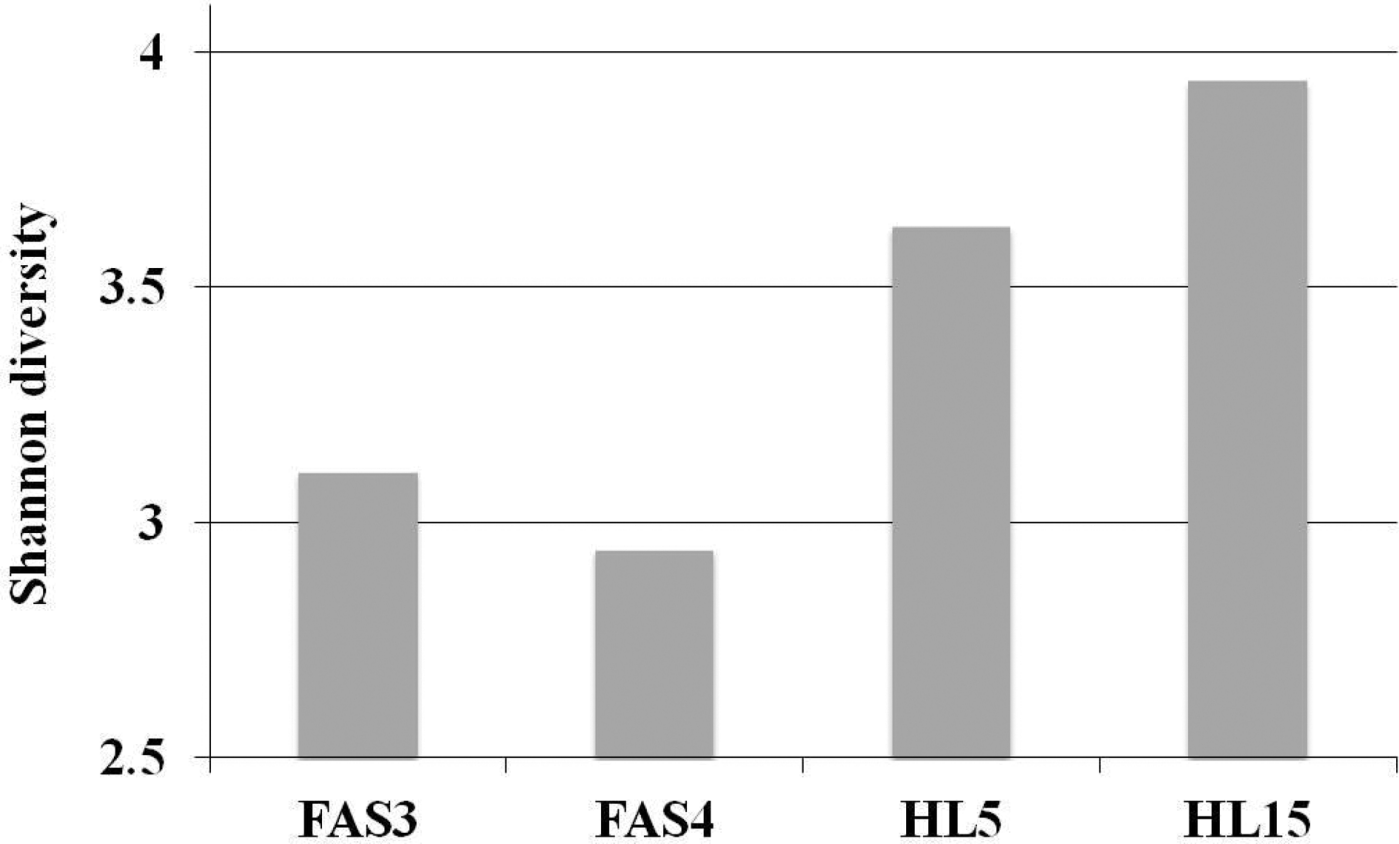
Shannon diversity (taxonomic rank: family) between the glacier-fed turbid FAS 3 and the clear FAS 4 lake in the Austrian Alps and the glacier-fed turbid HL 5 and clear HL 15 lake in the Himalayan mountains. Only amplicons are considered that were at least 95% similar to database entries; Faselfad lakes, FAS; Himalaya lakes, HL.

In the turbid FAS 3, OTUs were assigned to 34 different taxonomic families, and in FAS 4, we detected 28 different families (Fig. 3, Table S1,Supporting Information). Interestingly, in the HL lakes, the clear HL 15 harbored a larger number of families (*n* = 39) as HL 5 (*n* = 35). Accordingly, Shannon diversity in both HL lakes was higher than in the FAS lakes, and the clear HL 15 was more diverse than HL 5 (Fig. 4).

**Figure 5.**
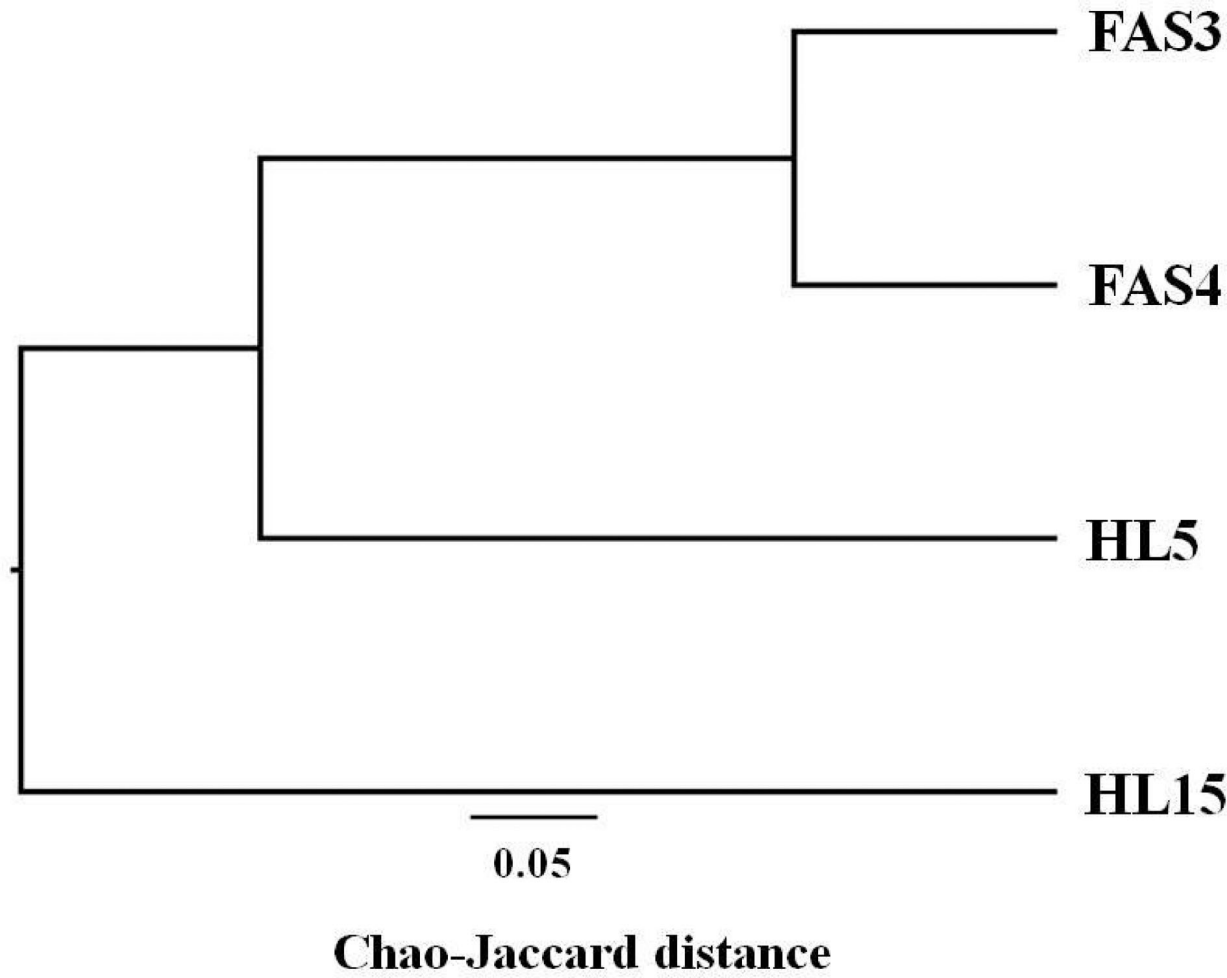
UPGMA clustering of Chao–Jaccard beta-diversity based on OTUs called at 97% sequence similarity (for details, see the section ‘materials and methods’). Lakes from Alps in Austria (FAS) are more similar to each other than to either of the two HL lakes regarding protistan (incl. fungi) community composition. Furthermore, similarity between the glacier-fed turbid and the clear lake in the Alps (FAS 3 and FAS 4, respectively) was notably higher than similarity between communities in the glacier-fed turbid and clear lakes in the Himalayan mountains (HL 5 and HL 15, respectively). The same pattern was observed when the analysis was conducted with taxonomic families detected in the four lakes (Fig. S5, Supporting Information).

Differences in community composition between the glacier-fed and the clear lakes in both geographic regions were confirmed by significant Fishers exact tests (*P* < 0.01). In addition, communities between the HL and the Alpine lakes were significantly different (Fishers exact tests, *P* < 0.01). Partitioning of diversity (Chao–Jaccard) shows that the two FAS lakes were more similar to each other regarding their protistan plankton communities than to either of the two HL lakes. Furthermore, the Chao–Jaccard distance among the FAS lakes was smaller than the distance among the HL lakes. This applies to both, analyses conducted with OTUs_97%_ obtained from all four lakes (Fig. 5) as well as to taxonomic families observed in the four samples (Fig. S5, Supporting Information).

In comparison to the FAS lakes, the network analysis shows that the HL lakes had notably more families of the Chloroplastida (10 vs 5) and the Stramenopiles (14 vs. 8), whereas alveolate (18 vs 16) and fungal families (5 in both cases) were in the same order of magnitude (Fig. 3). None of the fungal families was shared between the two geographic regions. The HL lakes additionally harbored one cercozoan, one telonemid and one choanomonad family that were not detected in the FAS lakes. On the other hand, one acanthoid family (Centrohelida) was exclusively detected in the FAS lakes.

The FAS lakes shared 24 families (Fig. 3), most of them assigned to the Alveolata (12 families) and Stramenopiles (6 families). Interestingly, fungi (five families) were exclusively detected in FAS 3. In total, ten families were unique to FAS 3 and only four to the clear one.

In total, 52 different families were detected in the two HL lakes, 22 of which were shared. The only choanomonad family was exclusively found in the turbid HL 5. Additionally, HL 5 harbored only three unique alveolate families, whereas in the clear HL 15 seven unique alveolate families were found. An overview about the families detected in each lake is given in Table S1 (Supporting Information).

## DISCUSSION

The few available studies focusing on microbial eukaryote plankton diversity in high mountain lakes have exposed only the tip of the iceberg of an as yet undiscovered microbial diversity in this type of extreme freshwater ecosystem (Félip *et al.*, 1999; Straškrabová *et al.*, 1999; Wille *et al.*, 1999; Sonntag, Summerer and Sommaruga 2011; Triadó-Margarit and Casamayor 2012). In our study, we found 3252 molecular OTUs called at 97% sequence similarity (OTUs97_%_, Table 2), blasting with 66 distinct taxonomic families. This exceeds previous investigations by far. However, we here note that translating molecular OTUs into taxonomical hierarchies such as morphospecies is very difficult (if not even impossible at this time). This is mainly because the genetic variability in taxonomic marker genes is not always taxonomically informative (Caron *et al.*, 2009; Nebel *et al.*, 2011b; Dunthorn *et al.*, 2012) and the accuracy of taxonomic assignments can be unsatisfying, specifically for short sequence fragments (Stoeck *et al.*, 2014). For further reasons, discussed in detail previously, we refer to Caron *et al.* (2009), Nebel *et al.* (2011b) and Stoeck *et al.* (2014). Therefore, to compare our molecular data with previous studies, we here discuss our taxonomically assigned OTUs preferably on a higher taxonomic level, namely the family rank, which is a relatively solid and reliable assignment rank for short V4 tags in microbial eukaryotes (Stoeck *et al.*, 2010; Dunthorn *et al.*, 2012).

The major taxon groups detected in the four lakes corroborate with those found in the gene-based study of Triadó-Margarit and Casamayor (2012) for lakes located in the Central Pyrenees, Spain. The authors found that most of the sequences belonged to the stramenopiles, alveolates and cryptophytes, but also to sequences of opisthokonts (fungi), chloroplastids, rhizaria, and a few katablepharids, euglenozoans, and *Telonema*. Although different molecular techniques have been applied in the latter survey, our study supports their taxonomic pattern (except for euglenozoans) because we also found that most of the sequences belong to the stramenopiles, alveolates and cryptophytes (Fig. 1).

In the HL lakes (Figs 2 and 3), we detected that >90% of the target sequences belong to the alveolates (>80% of which were Dinophyceae) and stramenopiles (>70% chrysophytes, i.e. Chrysophyceae and Synurophyceae). In the Alpine lakes, Chrysophyceae and Dinophyceae are key algal groups (Tolotti *et al.*, 2009) belonging to the most abundant taxa (e.g. Rott 1988). They can be indicators for environmental changes such as acidification or nutrient availability (Tolotti *et al.*, 2003). Therefore, a regular (molecular-based) time-series survey of these taxa may be important to assess environmental changes in high mountain lakes, which are extremely sensitive ecosystems (e.g. Catalan, Curtis and Kernan 2009; Modenutti *et al.*, 2013; Rogora *et al.*, 2013). Our study set the baseline and benchmark for such monitoring processes. Dinophyceae and Chrysophyceae seem well adapted to this extreme cold and nutrient-limited habitat type. This is coincident with their wide distribution even in the high arctic (e.g. Charvet *et al.*, 2012a; Charvet, Vincent and Lovejoy 2012b) and in glacial ice (García-Descalzo *et al.*, 2013). Survival strategies of Dinophyceae and Chrysophyceae are, for example, mixotrophy enabling them to adapt to low food and light supply by combining their heterotrophy and autotrophic nutrition modes (Stoecker 1999; Holen and Boraas 2009; Stoecker *et al.*, 2009; Charvet, Vincent and Lovejoy 2012b; McMinn and Martin 2013). Also recently, experiments have shown that mixotrophic chrysophycean species such as Dinobryon divergens, are less sensitive to the effect of glacial flour (Sommaruga and Kandolf, 2014). Furthermore, specialized life stages such as resting stages or cysts guarantee the survival under harsh environmental conditions. For example, nutrient shortage or low temperature are well known to induce cyst (resting stage) formation in many protists including dinoflagellates and chrysophytes, but also in ciliates (Kristiansen 1996; Foissner 2006, 2008; Mertens *et al.*, 2012). For instance, ∼24% of the freshwater dinoflagellates produce cysts (Mertens *et al.*, 2012) and siliceous cysts are also a substantial part of the chrysophyte life cycle (e.g. Sandgren 1991).

The Shannon diversity in the HL lakes exceeded that of the FAS lakes in the Austrian Alps (Fig. 4). For this high diversity in the HL lakes, several reasons could be taken into account, all of which, however, require in-depth investigations. One possibility for this higher diversity is input from atmospheric transport or from the catchment through precipitation in the Himalayan mountain region and/or nutrient availability. In general, atmospheric transport of microorganisms by air and dust is widespread among bacteria, fungal spores, protist cysts and pollen (e.g. Foissner 2006; Kellogg and Griffin 2006). Nepal and especially the Khumbu Valley region are strongly influenced by monsoon rainfalls. Particularly, from August to September, over 98% of the total annual precipitation falls in the Khumbu Valley (Lami *et al.*, 2010) so that probably more taxa are ‘washed out’ from the atmosphere or the catchment area. For example, typical Chlamydomonaceae (Chloroplastida) such as *Chlamydomonas nivalis* (‘Red Snow’) or *Chloromonas nivalis* live in snow and ice water and may be easily washed into a lake during snow and ice melting processes or rainfall (Hoham and Duval 2001; Remias *et al.*, 2010). Interestingly, Chlamydomonaceae (*C. raudensis*) are also found in permanently ice-covered polar lakes (Pocock *et al.*, 2004; Bielewicz *et al.*, 2011). In addition, Zhang *et al.* (2007) reported a higher diversity of cultivable bacteria in glacial ice in the Himalayan mountain region especially during the monsoon period. These authors showed evidence that this was associated with long transport of continental dust and marine air masses.

The diversity (Shannon diversity, Fig. 4) was higher in lakes with a lower nitrogen concentration (HL lakes; Table 1). Not surprisingly the nitrogen deposition and concentration is higher in the European Alps (Table 1) than in the Himalayan mountain region because of increased anthropogenic input (e.g. Rogora *et al.*, 2008). Consequently, nitrogen deposition can affect not only production and biomass of phytoplankton, but also their taxonomic composition as reviewed in Slemmons, Saros and Simon (2013). For example, certain taxa of the diatom family Fragilariaphycaea such as *Asterionella formosa* and *Fragilaria crotonensis* occurs in high abundances by nitrogen enrichment (e.g. Saros *et al.*, 2005). In our study, the most abundant diatoms belong to this family and were predominantly found in the FAS lakes (Suppl. Table 1) with higher nitrogen concentrations.

Therefore, the higher availability of reactive nitrogen in Alpine lakes could have been important in structuring the phytoplankton community in the FAS lakes.

In the HL lakes and in the FAS lakes, protistan community structures were significantly different between the glacier-fed turbid lakes and the clear ones (Figs 3 and 5). Less pronounced differences between the two FAS lakes compared to the differences between the two HL lakes may be attributed to the close vicinity of the FAS lakes, located in the same catchment area. Some years ago, the two FAS lakes were connected to the same glacier. This makes it reasonable to assume that they had a similar seed community of fungi and protists before FAS 4 lost connectivity to the glacier, giving rise to the evolution of a new plankton community. In contrast, the two HL lakes are located in different catchment areas and thus, these lakes may receive different input to maintain and support the corresponding plankton community structures.

Possible explanations for differences in plankton community structures in turbid and clear lakes are for example, the high loads of suspended particles (‘glacial flour’) from retreating glacier leading for example to a higher mortality of heterotrophic flagellates (Sommaruga and Kandolf 2014). Whereas, in clear alpine lakes, the potentially harmful UV-B can reach the lake bottom (Sommaruga and Psenner 1997; Laurion *et al.*, 2000; Sommaruga and Augustin 2006) and protists in such transparent habitats developed different strategies to protect themselves from high levels of incident solar radiation. Avoidance of high levels of solar radiation during solar noon or the synthesis or accumulation of photoprotective compounds and/or the presence of effective DNA repair mechanisms can be found among various planktonic organisms (e.g. Tilzer 1973; Sommaruga and Psenner 1997; Zagarese, Feldman and Williamson 1997; Alonso *et al.*, 2004; Sonntag, Summerer and Sommaruga 2007, 2011; Tartarotti *et al.*, 2014).

Worldwide, the retreat of glaciers is a given fact (e.g. Vaughan *et al.*, 2013) resulting in the emergence of numerous newborn proglacial lakes and loss in connectivity with glaciers. The latter process again is associated with major changes in lake physicochemical conditions in high mountain lakes. Selective mechanisms/behaviors as mentioned above are most likely major evolutionary forces governing shifts in plankton community structures when glacier-fed turbid lakes turn into clear ones after loss of glacier connectivity. In a first step, some turbid-lake taxa are probably eliminated from the original seed community, when turbid lakes shift to clear ones. This is because these taxa have low adaptive capabilities to survive in clear UVR-flooded waters. In a second step, succession of new taxa preferring clear-lake conditions complete the community shifts. To address this issue in detail future studies are needed focusing on the factors that affect community structures and ecosystem function(ing). Nevertheless, our study gives a first insight into microbial communities of glacier-fed and clear lakes revealing a high diversity and an important reservoir of largely unseen protistan diversity.

## SUPPLEMENTARY DATA

Supplementary data is available at FEMSEC online.

Supplementary data is available at FEMSEC online
