# Supplementary material for: High diversity of protistan plankton communities in remote high mountain lakes in the European Alps and the Himalayan mountains
Source: FEMS Microbiol Ecol. 2015 Jan 28;91(4):fiv010. doi: 10.1093/femsec/fiv010 (PMC4399440; doi:10.1093/femsec/fiv010)
Supplement: Supplementary data is available at FEMSEC online [file femsec_fiv010_index.html]

SUPPLEMENTARY DATA | FEMS Microbiology Ecology

## SUPPLEMENTARY DATA

**Files in this Data Supplement:**

- SUPPLEMENTARY DATA
