# Supplementary material for: High diversity of protistan plankton communities in remote high mountain lakes in the European Alps and the Himalayan mountains
Source: FEMS Microbiol Ecol. 2015 Jan 28;91(4):fiv010. doi: 10.1093/femsec/fiv010 (PMC4399440; doi:10.1093/femsec/fiv010)
Supplement: Supplementary data is available at FEMSEC online [file Supplementry_material.pdf]

Supporting information

Additional supporting information may be found in the online version of this article:

Table S1. Taxonomic assignment of V4-SSU rDNA amplicons detected in the turbid FAS 3 and the clear FAS 4 lakes in the Austrian Alps and the turbid HL 5 and clear HL 15 lakes in the Himalaya. Numbers in the table represent numbers of OTUs called at 97% sequence similarity that were assigned to a specific family, as well as numbers of V4 sequences (in brackets). Only amplicons are considered that were at least 95% similar to database entries; Faselfad lakes, FAS; Himalaya lakes, HL.

Fig. S1. Length distribution of sequences (basepairs; bp).

Fig. S2. Collapse of the number of OTUs with decreasing cluster threshold.

Fig. S3. Rarefaction curves for the different lakes (Faselfad lakes, FAS; Himalaya lakes, HL) based on 454 reads (only target sequences) using clustering thresholds between 90% and 100%. The curves show that sample and diversity saturation were proved for 97% cluster threshold in all lakes. Note different scales.

Fig. S4. Distribution of the similarities in percentage of identity with best BLAST hit. Note that most of the sequences (59.5%) have a similarity of at least 95% to database entries.

Fig. S5. UPGMA clustering of Chao-Jaccard beta-diversity based on taxonomic families detected in the lakes (for details see 'Materials and Methods'). Lakes from the Austrian Alps (Faselfad, FAS) are more similar to each other than to either of the

For Peer Review

two Himalayan lakes (HL) regarding protistan (incl. fungi) community composition. Furthermore, similarity between the glacier-fed turbid and the clear lake in the Alps (FAS 3 and FAS 4, respectively) was notably higher than similarity between communities in the turbid and clear lakes in the Himalaya (HL 5 and HL 15, respectively). Only amplicons are considered that were at least 95% similar to database entries.

For Peer Review

Table S1.

	Family	FAS3	FAS4	HL5	HL15
**ALVEOLATA**	Colpodellidae	1 (2)	1 (7)	1 (1)	0 (0)
	Platyophryidae	0 (0)	0 (0)	0 (0)	1 (3)
	Mesodiniidae	4 (155)	2 (25)	1 (35)	0 (0)
	Lacrymariidae	0 (0)	0 (0)	0 (0)	1 (5)
	Spathidiidae	5 (92)	0 (0)	0 (0)	1 (4)
	Tracheliidae	1 (1)	10 (3944)	0 (0)	0 (0)
	Trachelophyllidae	0 (0)	0 (0)	0 (0)	2 (27)
	Tetrahymenidae	0 (0)	0 (0)	0 (0)	1 (3)
	Opisthonectidae	1 (3)	0 (0)	0 (0)	0 (0)
	Vorticellidae	0 (0)	0 (0)	0 (0)	1 (41)
	Urotrichidae	0 (0)	0 (0)	2 (6)	7 (130)
	Halteriidae	0 (0)	0 (0)	1 (6)	1 (4)
	Oxytrichidae	0 (0)	0 (0)	0 (0)	1 (3)

For Peer Review

	Chytriodiniaceae	0 (0)	1 (13)	0 (0)	0 (0)
	Gymnodiniaceae	136 (1904)	143 (55877)	38 (60294)	25 (61811)
	Polykrikaceae	1 (1)	1 (8)	0 (0)	0 (0)
	Lophodiniaceae	3 (15)	1 (1)	3 (19)	0 (0)
	Heterocapsaceae	0 (0)	1 (5)	0 (0)	0 (0)
	Peridiniaceae	136 (4455)	127 (11428)	57 (13490)	37 (15020)
	Pfiesteriaceae	38 (1009)	38 (2243)	9 (2719)	10 (3320)
	Phytodiniaceae	1 (2)	1 (1)	2 (12)	2 (28)
	Prorocentraceae	1 (4)	3 (9)	0 (0)	0 (0)
	Symbiodiniaceae	17 (81)	29 (2316)	1 (2424)	1 (2492)
	Oodiniaceae	165 (7103)	153 (19689)	29 (19808)	7 (19848)
**FUNGI**	Coniochaetaceae	0 (0)	0 (0)	0 (0)	1 (3)
	Agaricaceae	2 (63)	0 (0)	0 (0)	0 (0)
	Tricholomataceae	2 (64)	0 (0)	0 (0)	0 (0)
	Tulasnellaceae	1 (12)	0 (0)	0 (0)	0 (0)

For Peer Review

	Polyporaceae	2 (110)	0 (0)	0 (0)	0 (0)
	Fomitopsidaceae	1 (12)	0 (0)	0 (0)	0 (0)
	mitosporic Cystofilobasidiales	0 (0)	0 (0)	1 (7)	1 (1)
	Filobasidiales incertae sedis	0 (0)	0 (0)	0 (0)	1 (6)
	Tremellales incertae sedis	0 (0)	0 (0)	3 (88)	0 (0)
	Leucosporidiaceae	0 (0)	0 (0)	1 (22)	0 (0)
**STRAMENOPILES**	Achnanthidiiaceae	0 (0)	0 (0)	1 (4)	1 (2)
	Cymbellaceae	8 (1249)	2 (8)	3 (4)	2 (88)
	Gomphonemataceae	0 (0)	0 (0)	1 (3)	0 (0)
	Coscinodiscaceae	0 (0)	0 (0)	0 (0)	1 (8)
	Thalassiosiraceae	0 (0)	1 (4)	1 (3)	0 (0)
	Fragilariaceae	41 (17067)	21 (17277)	8 (17288)	4 (17643)
	Naviculaceae	1 (1)	1 (3)	7 (1364)	3 (9)
	Chromulinacea	6 (107)	4 (157)	2 (24)	0 (0)
	Dinobryaceae	7 (39)	9 (280)	16 (2434)	15 (3575)

For Peer Review

	Paraphysomonadaceae	0 (0)	0 (0)	1 (3)	1 (2)
	Pedinellaceae	1 (1)	1 (13)	0 (0)	0 (0)
	Monodopsidaceae	1 (9)	0 (0)	1 (2)	0 (0)
	Saprolegniaceae	0 (0)	0 (0)	0 (0)	1 (4)
	Mallomonadaceae	0 (0)	0 (0)	0 (0)	1 (7)
	Synuraceae	0 (0)	0 (0)	0 (0)	1 (32)
**CHLOROPLASTIDA**	Chlamydomonadaceae	5 (23)	1 (51)	6 (85)	26 (722)
	Chlamydomonadales incertae sedis	0 (0)	0 (0)	1 (10)	1 (22)
	Chlorococcaceae	0 (0)	0 (0)	0 (0)	1 (56)
	Dunaliellaceae	0 (0)	0 (0)	1 (2)	1 (43)
	Neochlorosarcina incertae sedis	1 (6)	0 (0)	0 (0)	1 (7)
	Hydrodictyaceae	0 (0)	1 (5)	0 (0)	0 (0)
	Sphaeropleaceae	0 (0)	0 (0)	1 (4)	0 (0)
	Sphaeropleales incertae sedis	3 (233)	2 (16)	1 (45)	1 (1)
	Chlorellaceae	1 (1)	3 (11)	1 (9)	2 (6)

For Peer Review

	Oocystaceae	0 (0)	0 (0)	1 (8)	0 (0)
	Coccomyxaceae	0 (0)	0 (0)	0 (0)	2 (3)
**CHOANOMONADA**	Codonosigidae	0 (0)	0 (0)	1 (4)	0 (0)
**CERCOZOA**	Thaumatomastigidae	1 (2)	1 (1)	1 (7)	1 (28)
**OTHERS**	Acanthocystidae	1 (4)	0 (0)	0 (0)	0 (0)
	Telonema incertae sedis	0 (0)	0 (0)	0 (0)	1 (10)
	Cryptomonadaceae	44 (13597)	30 (526)	3 (48)	0 (0)
	Chroomonadaceae	1 (2)	1 (1)	1 (71)	1 (5)

For Peer Review

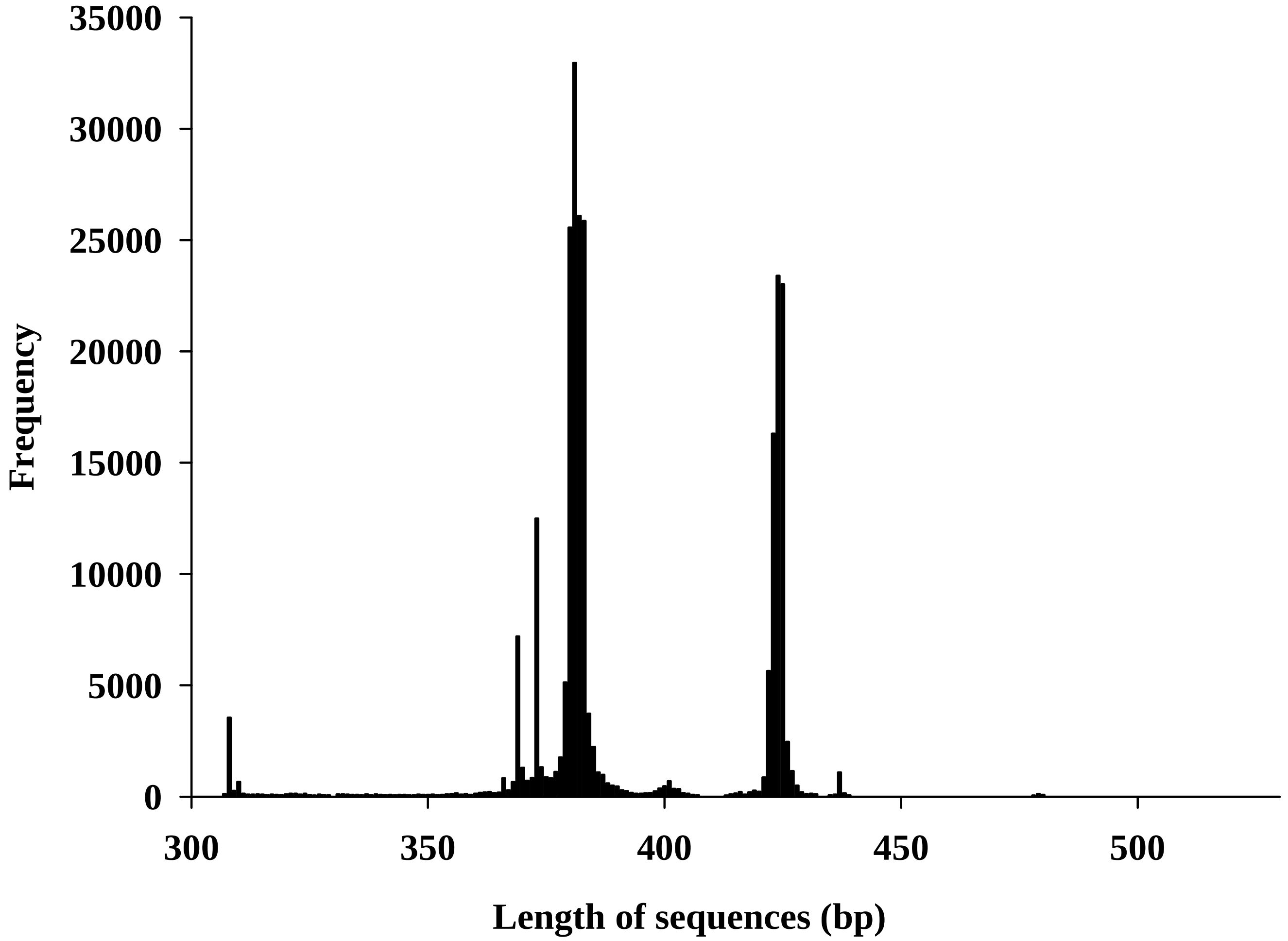
35000
30000
25000
20000
15000
10000
5000
0
Frequency
300
350
400
450
500
Length of sequences (bp)

Fig. S1.

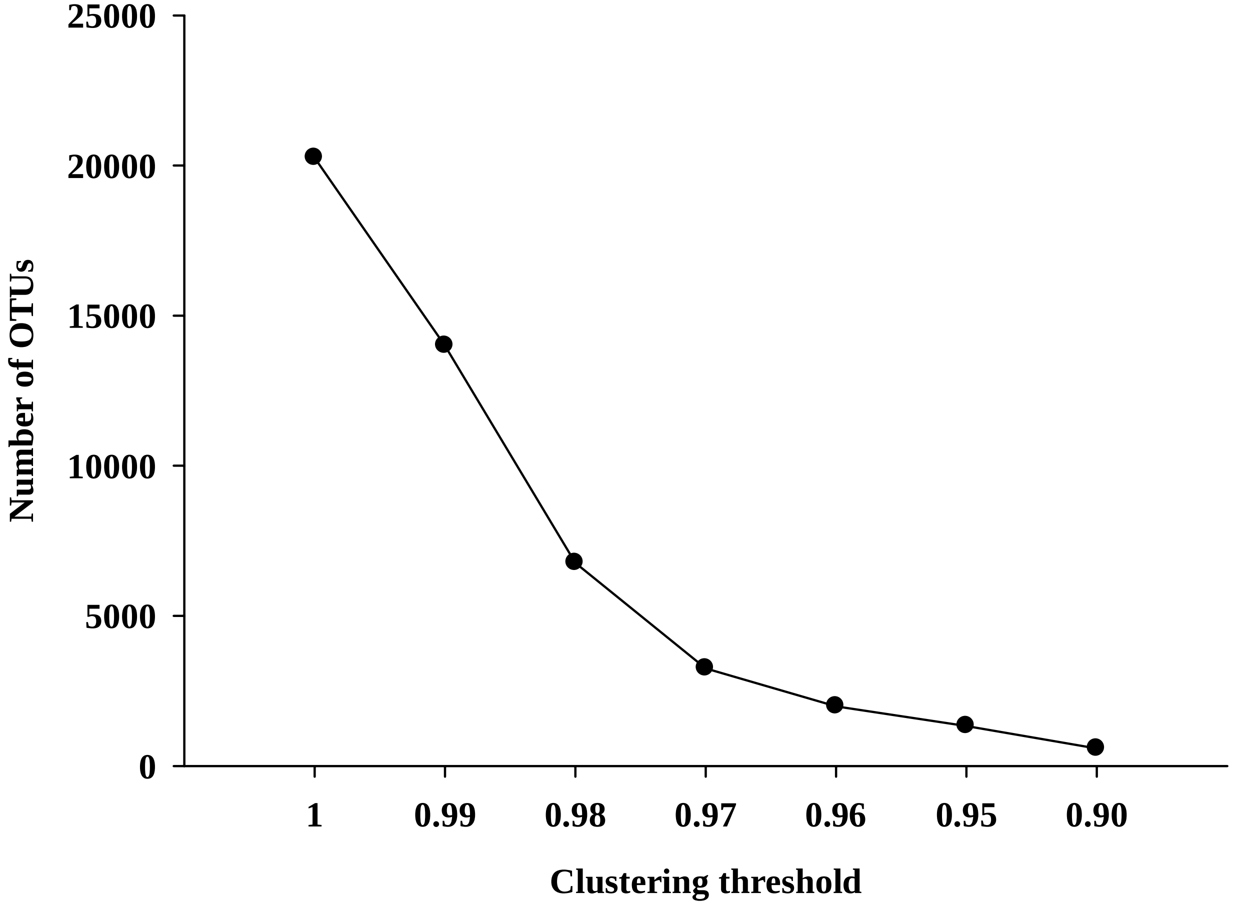
25000
20000
15000
10000
5000
0
Number of OTUs
1
0.99
0.98
0.97
0.96
0.95
0.90
Clustering threshold

Fig. S2.

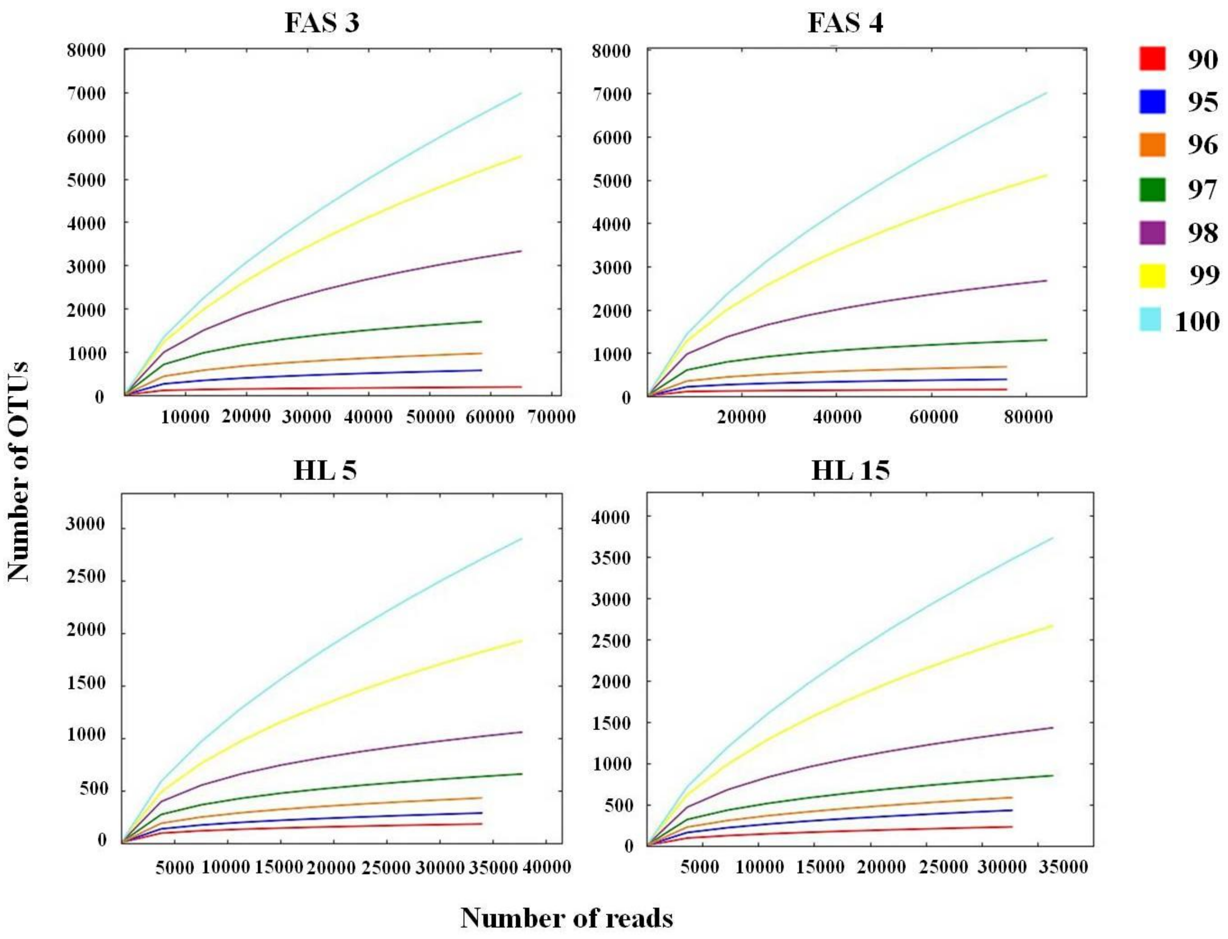
FAS 3
FAS 4
HL 5
HL 15
Number of OTUs
Number of reads
90
95
96
97
98
99
100
8000
7000
6000
5000
4000
3000
2000
1000
0
10000 20000 30000 40000 50000 60000 70000
20000 40000 60000 80000
3000
2500
2000
1500
1000
500
4000
3500
5000 10000 15000 20000 25000 30000 35000 40000
5000 10000 15000 20000 25000 30000 35000

Fig. S3.

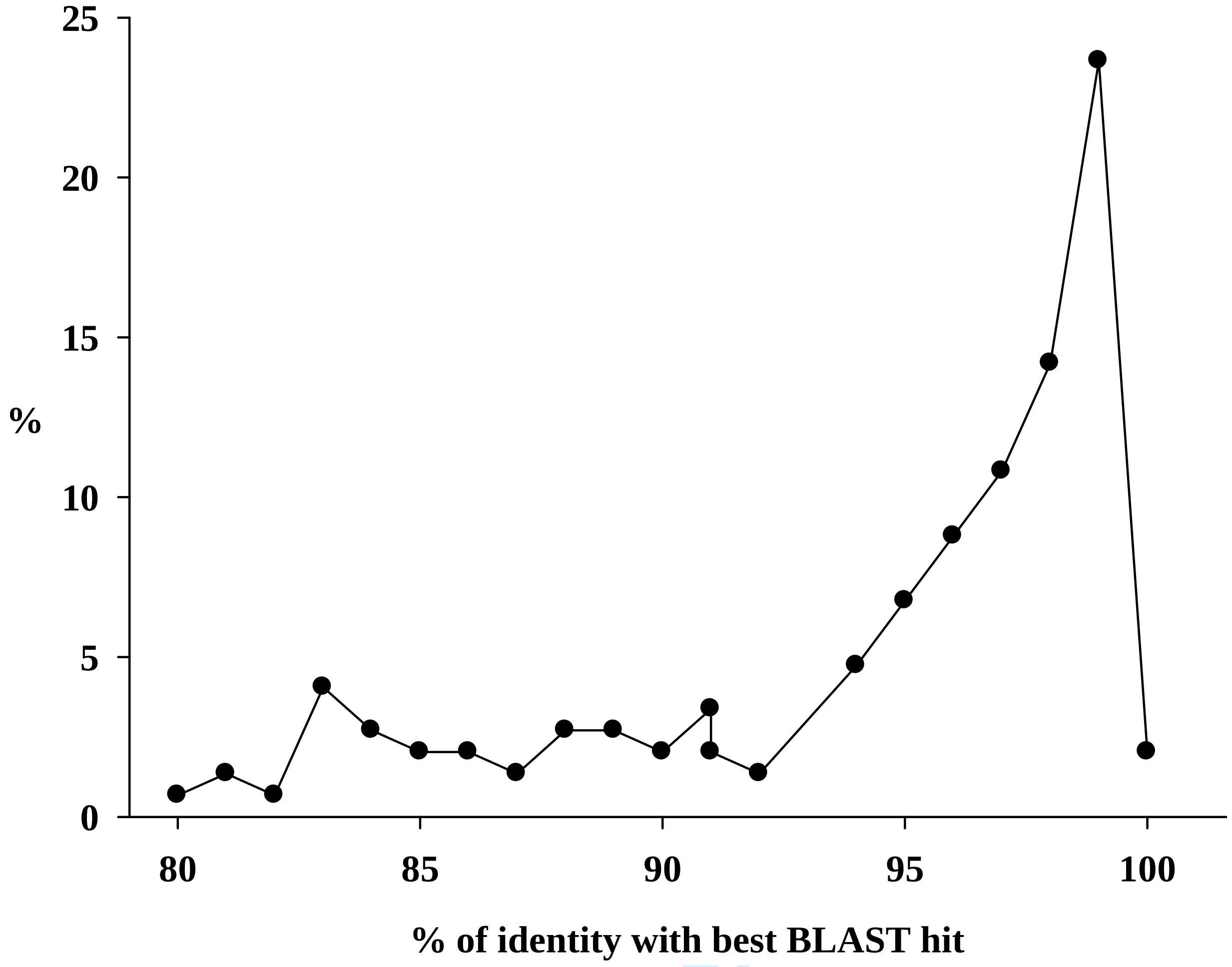

Fig. S4.

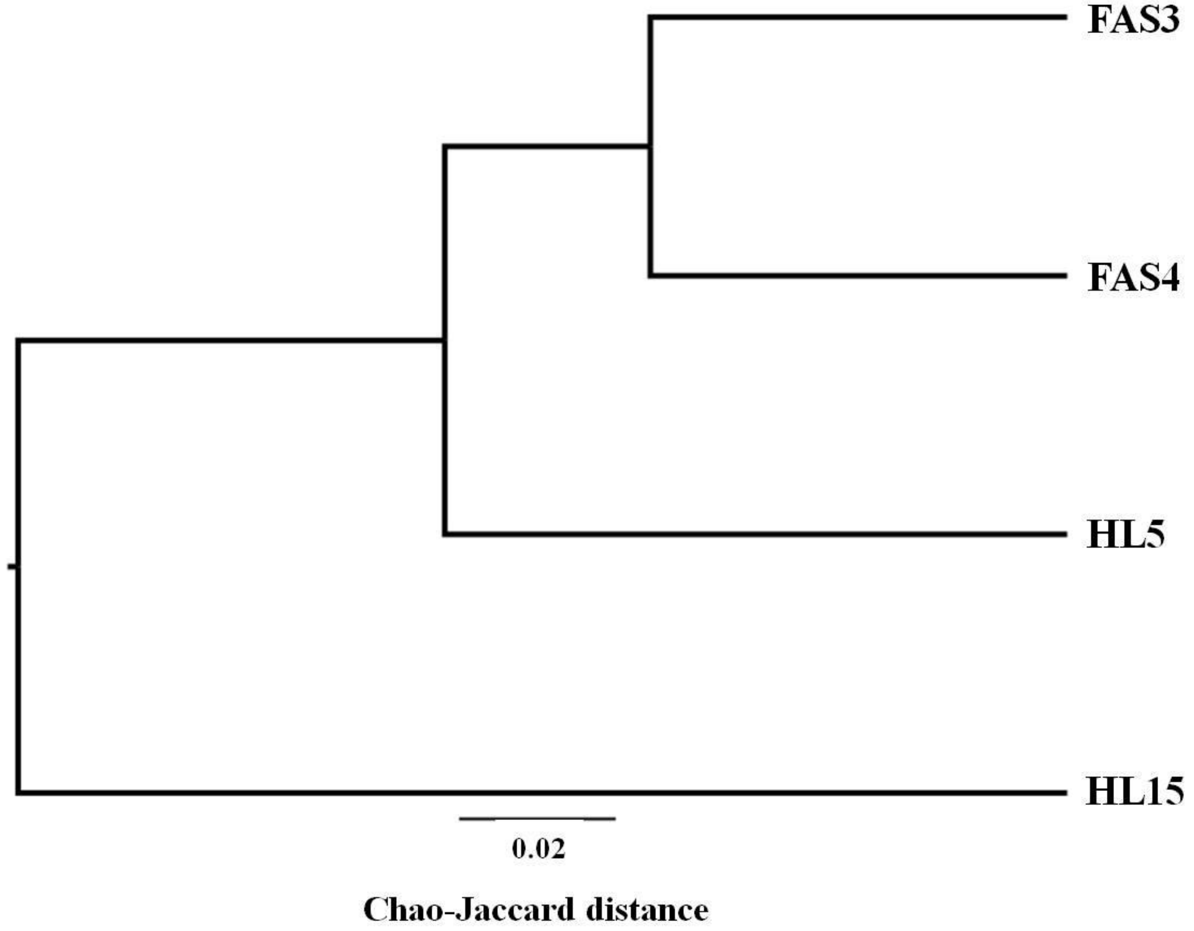

Fig. S5.
